# Prevalence and factors associated with transfusion-transmissible infections (HIV, HBV, HCV and Syphilis) among blood donors in Gabon: Systematic review and meta-analysis

**DOI:** 10.1371/journal.pone.0307101

**Published:** 2024-08-19

**Authors:** Christian Mangala, Denis Maulot-Bangola, Alain Moutsinga, Serge Christian Okolongo-Mayani, Gwladys Esmeralda Matsomo-Kombet, Mireille Moundanga, Christian Mombo-Maganga, Alfred Keith Felix Mabika-Obanda, Joseph Fokam

**Affiliations:** 1 National Public Health Laboratory, Libreville, Gabon; 2 Peyrie Medical Center, Libreville, Gabon; 3 Chantal BIYA International Reference Center for Research on HIV/AIDS Prevention and Management, Yaoundé, Cameroon; 4 Doctoral Training Unit of School of Health Sciences, Catholic University of Central Africa, Yaoundé, Cameroon; U.S. Food and Drug Administration, UNITED STATES OF AMERICA

## Abstract

**Background:**

Transfusion-transmissible infections (TTIs) remain a major public health problem in countries with limited resources, particularly in Gabon. Complete information on the prevalence in Gabon of the main TTIs among blood donors is still lacking in the national context. The purpose of this systematic review and meta-analysis was to determine the prevalence and factors associated with TTIs among blood donors in Gabon.

**Methods:**

This systematic review and meta-analysis was reported in accordance with the PRISMA 2020 guidelines. It was the result of data from several comprehensive studies published between 2014 and 2022, the purpose of which focused on the prevalence and factors associated with TTIs among blood donors in Gabon. The quality of the articles was assessed using the Joanna Briggs Institute critical appraisal checklist for studies reporting prevalence data. The overall prevalence of TTIs among blood donors was determined using the random effects model. Heterogeneity between studies was assessed using I^2^ statistics. Publication bias was assessed by visual inspection of the funnel plot and Egger’s statistics.

**Results:**

A total of 175,140 blood donors from the nine eligible studies were admitted to this study. The combined prevalence of HIV, HBV, HCV and syphilis obtained in the random effects model was 3.0%, 6.0%, 4.0% and 3.0%, respectively. Moreover, being a male blood donor and aged between 25 and 44 years was significantly associated with HBV infection and being a female blood donor and aged 35 years and over was significantly associated with HIV infection. Family or replacement blood donors had a high infection burden for all four TTIs of study.

**Conclusion:**

The overall prevalence of transfusion-transmissible infections remains high in the country’s blood banks. Improving current prevention (selection criteria) and screening strategies may be necessary in a global approach.

## Introduction

Blood transfusion is intended to resolve problems linked to a drop in labile blood products (LBP) in patients suffering from severe anemia, a traffic accident or during surgery. But some infections can be transmitted by blood transfusion. These transfusion-transmissible infections (TTIs) are an ongoing threat in blood transfusion in resource-limited countries, particularly those in sub-Saharan Africa **[1–4].** These transfusion-transmissible infections are due to certain hematogenous germs which can be viruses, bacteria, parasites and prions. But the germs most encountered in TTIs in blood donors are the human immunodeficiency virus (HIV), hepatitis B and C viruses (HBV and HCV), and Syphilis, because of their levels high prevalence rates **[5]**.

Several studies conducted on transfusion-transmissible infections have shown that the prevalence of TTIs varies between resource-limited and high-income countries. Some authors have shown that the prevalence of TTIs (HIV, HBV, HCV and Syphilis) in high-income countries is very low. In particular those of HIV, HBV, HCV and Syphilis which were respectively 0.002%, 0.02%, 0.007% and 0.02%. While in resource-limited countries, particularly sub-Saharan African countries, the prevalence of TTIs in these countries was high, notably HIV, HBV, HCV and Syphilis was respectively 0.70%, 2.8%, 1.0% and 0.90%. This difference reflects the prevalence of TTIs in the population of these two categories of countries **[6–8].** The situation of TTIs in Gabon is almost identical to that of countries with limited resources because some previous studies carried out on TTIs showed that the prevalence of TTIs was worrying **[9].**

Developed countries have succeeded in reducing the incidence of TTIs. However, blood safety continues to be a major issue in developing countries **[10,11]**.

The circulation of hematogenous pathogens in the transfusional settings must challenge blood bank managers on a daily basis to strengthen donation screening strategies. But also to update information on transfusion-transmissible infections in order to improve and strengthen the monitoring of pathogens circulating in the donor. Similarly, haemovigilance should be a central concern of any blood bank manager **[12–14]**.

In sub-Saharan African countries, data on transfusion-transmissible infections must be updated daily to better ensure optimal surveillance of TTIs. In Gabon, the documentation on TTIs is insufficient to ensure optimal monitoring of the infectious agents circulating among blood donors. But this must be accompanied by a strengthening of current donation screening strategies for better production of reliable data allowing good decisions to be made on the surveillance of TTIs in the country **[15–18]**.

Monitoring transfusion-transmissible infections in blood donors can provide a better understanding of the epidemiology of these TTIs. This can also strengthen prevention and screening strategies to ensure a safe blood supply and also better manage this infectious burden. Strengthening documentation on the prevalence of the main transfusion-transmissible infections (HIV, HBV, HCV and syphilis) which circulate among Gabonese blood donors. It is with this in mind that we conducted this study focused on a systematic review and a meta-analysis whose objective is to determine the prevalence and factors associated with transfusion-transmissible infections among blood donors in Gabon.

## Methods

### Study design

This study resulted from a systematic review with meta-analysis of summarized data from several studies focused on the prevalence of the main transfusion-transmissible infections (HIV, HBV, HCV and Syphilis) among blood donors in the Gabonese context. Indeed, we carried out a meta-analysis on the data retrieved from the full articles. These were obtained by reading the article most often presenting the results of all the participants included in each study.

### Registration of protocol design

Preferred Reporting Items for Systematic reviews and Meta-Analysis (PRISMA) protocols were used for this review **[19]**. The protocol had been registered in PROSPERO with a registration number: CRD42023407124.

### Search strategy

Studies on the prevalence of transfusion-transmissible infections (HIV, HBV, HCV and syphilis) among blood donors in the Gabonese transfusional settings were systematically searched January 3, 2023 in the various databases, namely PubMed, Embase and Google Scholar. To search the data for this study, we developed search terms in accordance with the Medical Subject Headings thesaurus (MeSH) and Boolean operators (AND, OR) were used to search the articles. The following terms in combination with key terms, such as: “Transfusion-transmissible viral hepatitis”, “Prevalence of transfusion-transmissible infections”, “Prevalence”, “Risk factors”, “Transfusion-transmissible infections”, “HIV”, “Hepatitis C virus”, “Syphilis”, “Hepatitis B virus”, “Blood donor”, “Blood transfusion” and “Gabon” were used.

### Selection criteria

**Fig 1:**
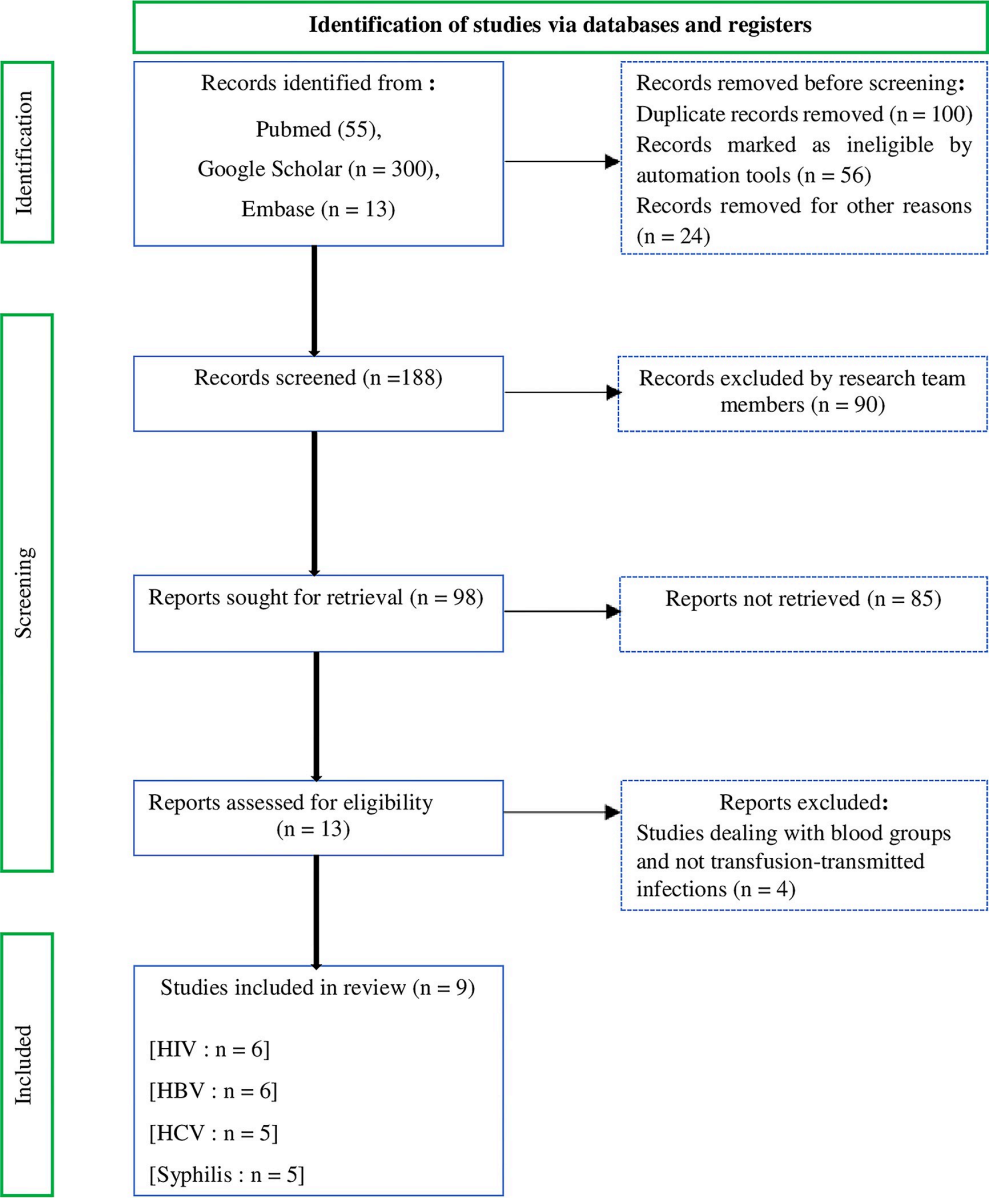
Flow diagram.

This study was conducted and written in accordance with the guidelines of PRISMA of the year 2020 **[19,20]**. The articles were selected independently by two groups composed of three authors each: group N°1 (CM, SCOM, AM) and group N°2 (DMB, MM, CMM). The full texts of the studies were assessed by the two groups of authors and in case of discrepancies, the third group of authors (AKFMO, JF, GEMK) was responsible for removing the ambiguity. And concerning the three hundred and sixty-eight studies that were retrieved from the various databases, nine studies met the eligibility criteria, namely the published studies having Gabonese blood donors as participants, having determined the prevalence of one or more TTIs (HIV, HBV, HCV and syphilis), having been published between 2014 and 2022, and the methodology based on 3rd or 4th generation serological tests. All studies published in English and French were included. On the other hand, for non-inclusion criteria, three hundred and fifty-nine articles were excluded for various reasons such as studies lasting more than 9 years, studies having been carried out outside the Gabonese transfusional settings, studies with a size of less than 300 participants, studies with inconsistent and non-usable results, and other reasons not allowing us to associate these studies with our meta-analysis study (Fig 1).

### Quality assessment

The methodological quality of the included studies was assessed using the Joanna Briggs Institute critical appraisal checklist for studies reporting prevalence data **[21,22]**. This critical appraisal checklist consisted of nine questions that inquired about study size (≥ 300), study setting information, methods used, participants (donor only) and other information related to the purpose of the study contributed to the evaluation of the different studies selected by a scoring system. Studies were considered to be of high quality for a score between 7–9, of moderate quality for a score between 4–6 and of poor quality for a score less than or equal to 3. The quality of the studies was independently assessed by two members (CM and DMB) and checked by a third member of the team (JF).

### Data abstraction

All data from eligible studies were extracted simultaneously by two researchers (CM and DMB) and then they checked for inconsistencies. Data extraction was performed using a standardized form in Microsoft Excel. Data collected included first author name, year of publication, sample size, study design, study region, baseline prevalence of each study, factors associated with each TTI, sociodemographic data, and serological status. Then, all abstractions and calculations were verified by all members of the research team. And for more information on included articles, authors were contacted where necessary. However, studies for which data were not obtained or calculated were simply excluded from the systematic review and meta-analysis.

### Statistical analysis

The prevalence of people positive for TTIs among blood donors was taken as the size of the effect. RevMan software version 5 was used for data management of the studies included for the meta-analysis and to present the results in graphical form (Forest plot). STATA software version 18 (Stata Corp LLC, Texas, USA) for statistical analysis was used in this study. The extent of heterogeneity between the included studies was determined quantitatively by the I^2^ statistical test **[23]**. The values of I^2^ made it possible to show whether the heterogeneity was low (25%), medium (50%) or high (≥ 75%). The extent of heterogeneity was determined by the P-value of the I^2^ statistics and a P- value < 0.05 was considered significant heterogeneity. The random-effects model was used for the medium and high levels of heterogeneity between the included studies **[24]**. Additionally, publication bias was assessed using the visual funnel chart test and Egger’s statistics. The random-effects model was used to account for the combined prevalence of TTIs among blood donors. ORs were determined for each of the reported variables (risk factors) and pooled in a meta-analysis. The 95% confidence interval and P-value were determined to estimate the association between risk factors and each TTI. The results were presented in the form of text and graphics.

## Results

### Socio-demographic characteristics of blood donors

The literature search found 368 articles, 9 of which were declared eligible and included in this systematic review and meta-analysis study (Fig 1). A total of 175,140 blood donors were included in the study, 78.1% were men and 21.9% women. The representative age groups were between 18–24, 25–34, 35–44 and ≥ 45 years, with a percentage of 34.6%, 44.5%, 16.1% and 4.8% respectively. Family donations represented 64.5% of blood bank donations (Table 1). Of 175,140 blood donors included in the study, 15,473 blood donors tested positive for one of the TTIs either by the ELISA test or the Determine® test (Table 2).

**Table 1:** Socio-demographic data extracted from included studies.

Variables	N	%
**Sex** Male Female	136,738	78.1
	38,402	21.9
**Age** 18–24 25–34 35–44 ≥ 45	60,671	34.6
	77,900	44.5
	28,243	16.1
	8,326	4.8
**Type of blood donor** Volunteer Family	62,140	35.5
	113,000	64.5

**N** : Number **%** : Percentage.

**Table 2:** Characteristics of the studies included in the systematic review and meta-analysis of TTIs in blood donors.

Author	Year	Origin	Type of study	Study size	HIV HBV HCV Syphilis				Test used
					HIV reactive	HBV reactive	HCV reactive	Syphilis reactive	
Rerambiah et al [25]	2014	Libreville	Retrospective	41,074	1,269	2,547	2,481	N/A	ELISA
Tonda et al [26]	2017	Koula-Moutou	Retrospective	614	8	20	30	10	Determine^TM^ SD Bioline
Eko Mba et al [27]	2017	Libreville	Retrospective	27,210	925	N/A	327	871	ELISA
Eko Mba et al [28]	2018	Libreville	Retrospective	69,862	N/A	5,083	N/A	N/A	ELISA
Bisseye et al [29]	2018	Koula-Moutou	Retrospective	5,706	177	337	354	188	Determine^TM^ SD Bioline
Ngassaki-Yoka et al [30]	2018	Libreville	Retrospective	4,744	52	80	24	76	ELISA
Bisseye et al [31]	2019	Libreville	Retrospective	20,651	N/A	N/A	N/A	496	ELISA
Mangala et al [32]	2021	Libreville	cross-sectional	3,669	30	N/A	N/A	N/A	ELISA
Maulot-Bangola et al [33]	2021	Libreville	cross-sectional	1,610	N/A	88	N/A	N/A	ELISA

**N/A** : Not available.

### Combined prevalence of TTIs (HIV, HBV, HCV and syphilis)

**Fig 2:**
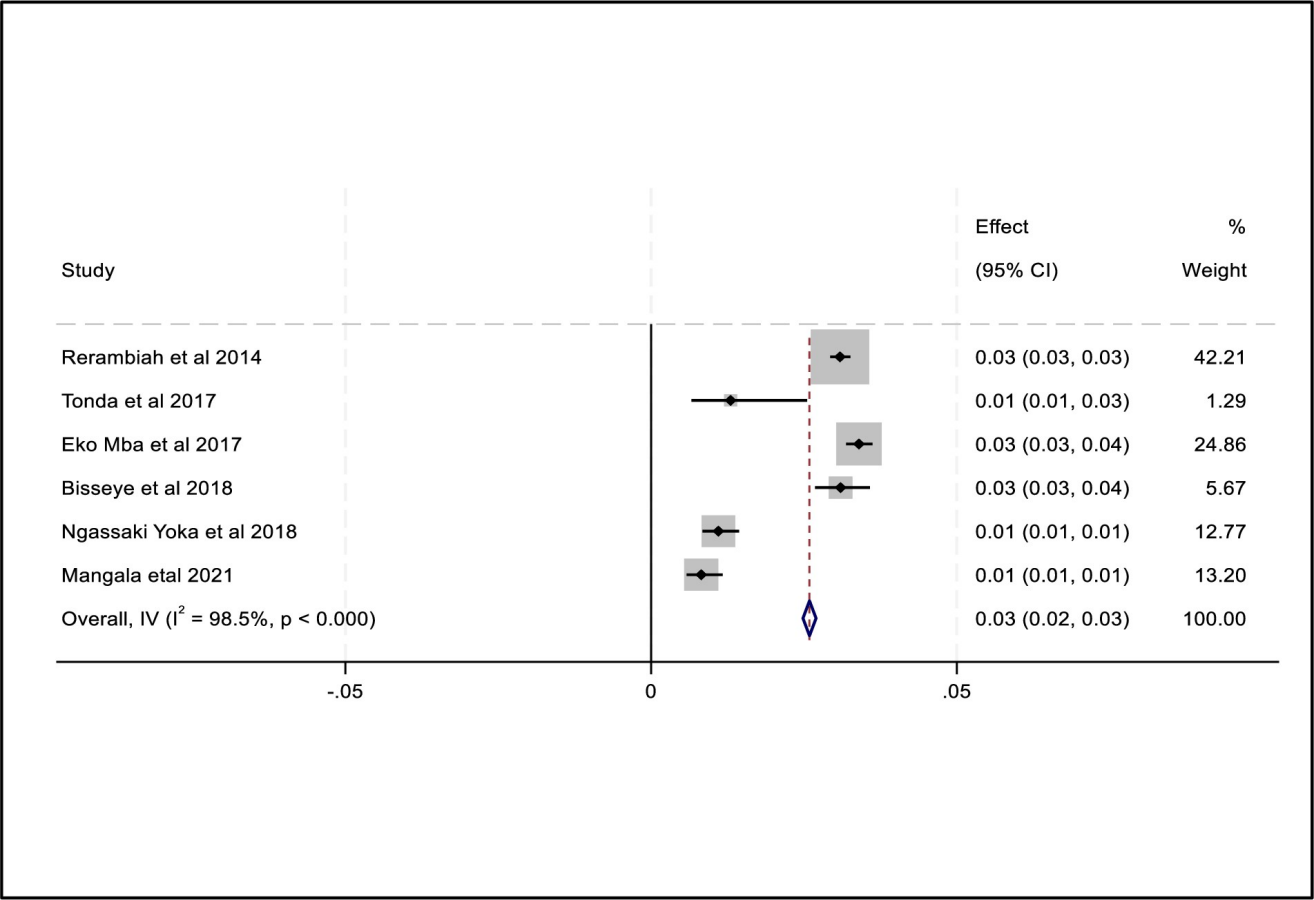
Forest plot of pooled HIV prevalence.

**Fig 3:**
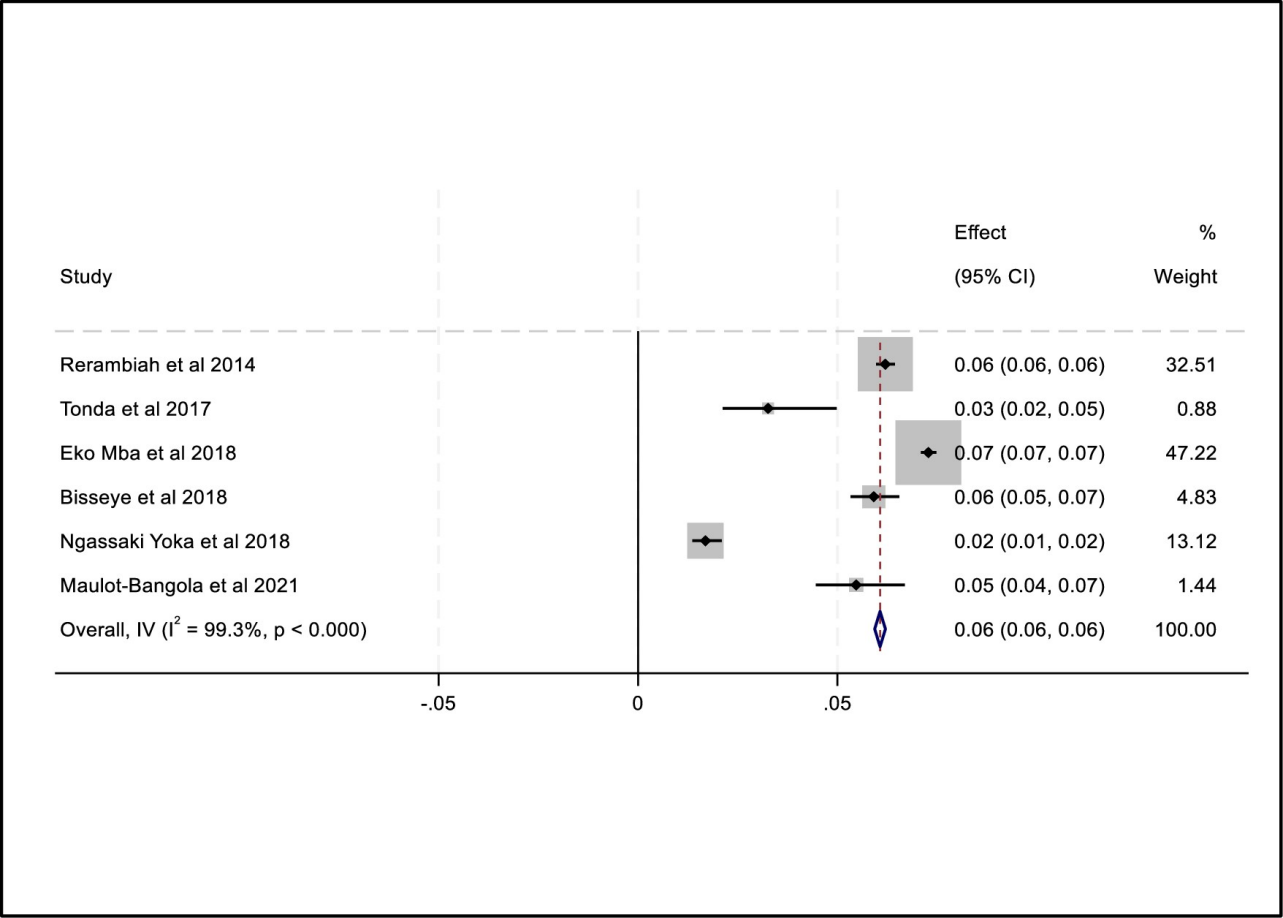
Forest plot of pooled HBV prevalence.

**Fig 4:**
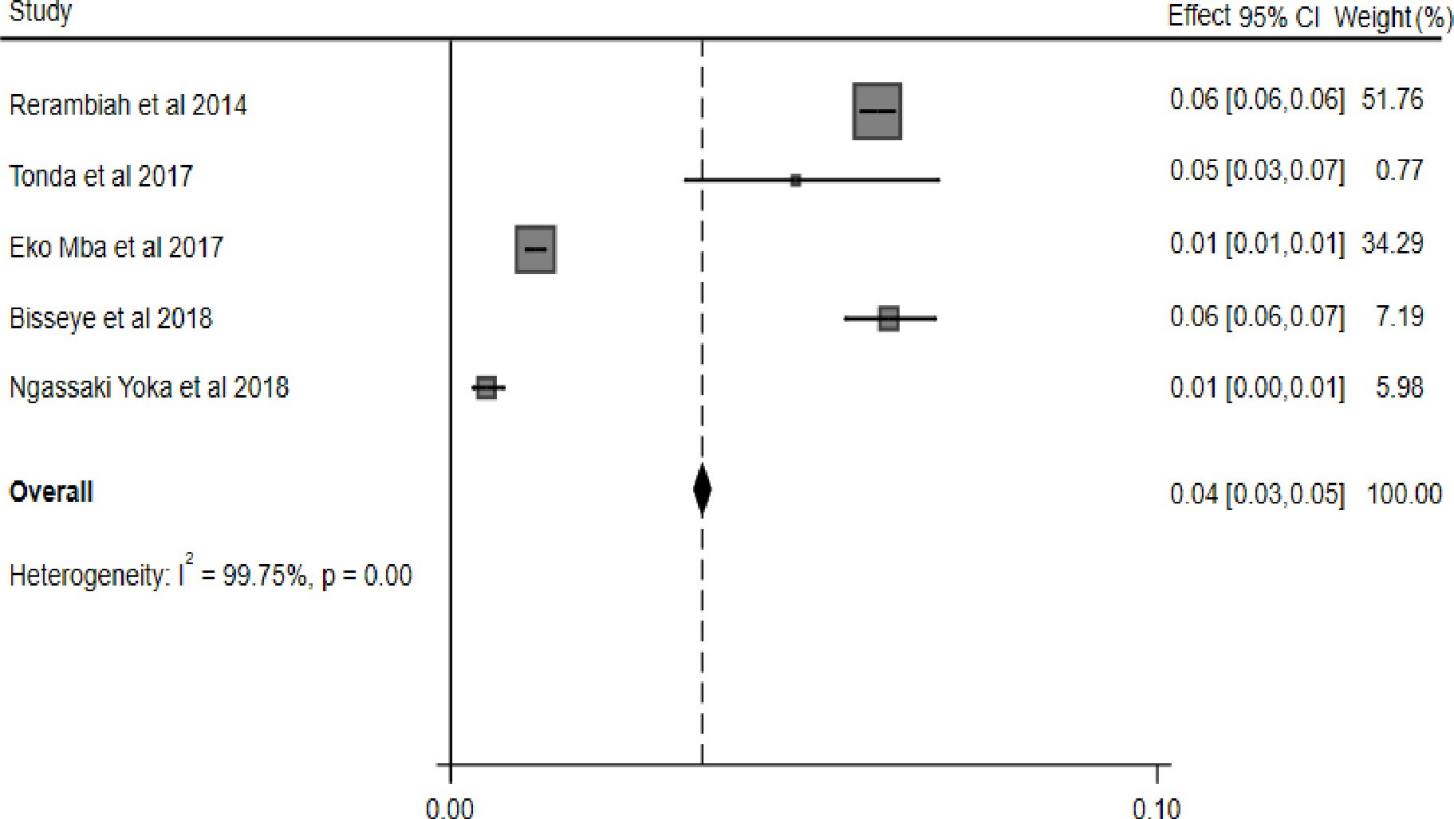
Forest plot of pooled HCV prevalence.

**Fig 5:**
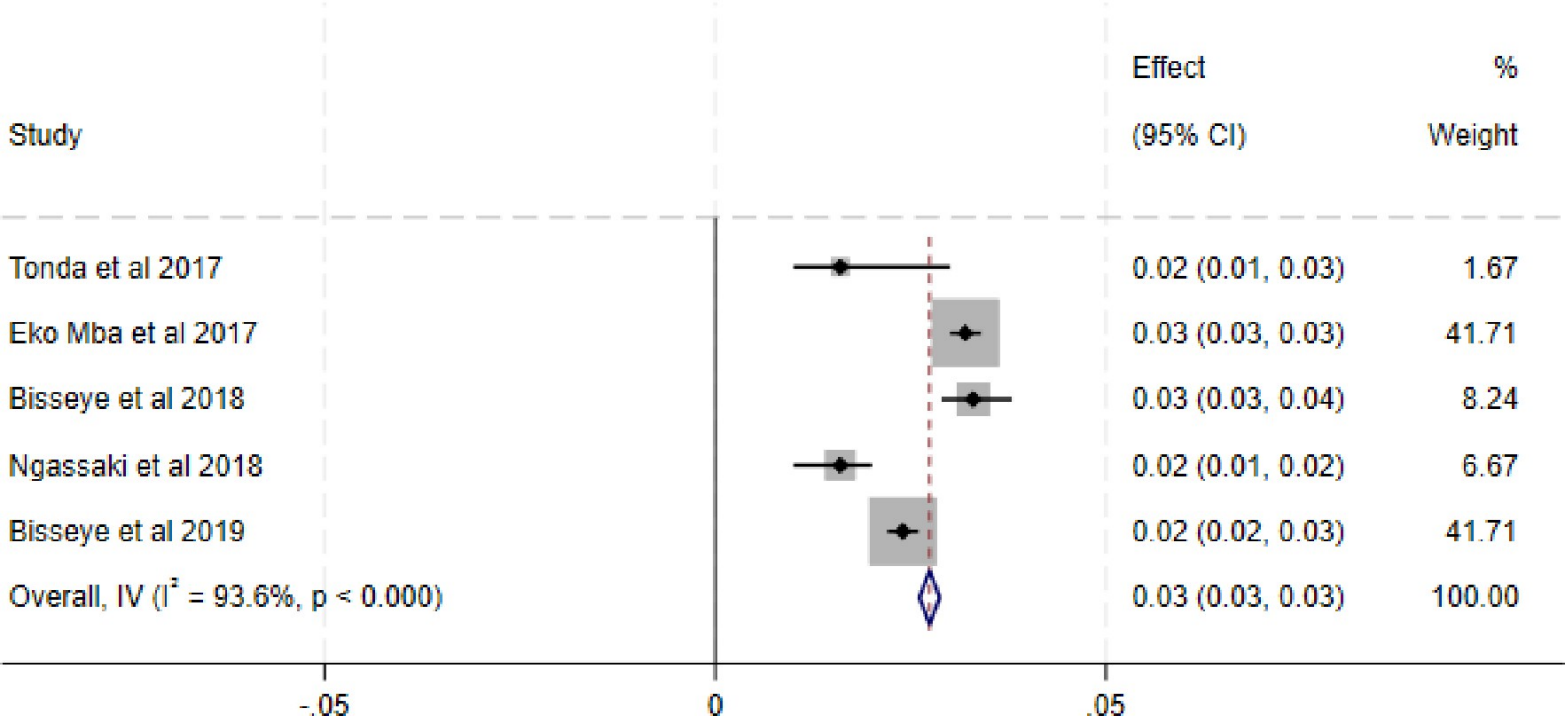
Forest plot of pooled Syphilis prevalence.

The combined prevalence of the four transfusion-transmissible infections among blood donors namely HIV, HBV, HCV and syphilis was 3.0%, 6.0%, 4.0% and 3.0% respectively. Subgroup analysis revealed that there were considerable variations in the estimate of the combined prevalence of HIV, HBV, HCV and syphilis between studies conducted in the Gabonese region. We did not find any obvious publication bias in the included studies to determine the overall prevalence of each TTI i.e. HIV, HBV, HCV and Syphilis using funnel plots (S1–S8 Figs) and the regression based on the Egger test (*P = 0*.*9314*; *P = 0*.*1840*; *P = 0*.*4357* and *P = 0*.*9001* respectively). The random-effects model was used and the heterogeneity value of the included studies for HIV was I^2^ = 98,5%, Q = 330.2 *P = 0*.*0001* (Fig 2). The heterogeneity of the included studies for HBV in the presence of the random effects model was I^2^ = 99,3%, Q = 770 with a significant P-value (*P = 0*.*0001*) (Fig 3). Similarly, the heterogeneity value of the included studies for HCV was I^2^ = 99.75%, Q = 1611.9 *P = 0*.*001* (Fig 4). And for syphilis, the heterogeneity of the included studies was I^2^ = 93.6% Q = 153.7 *P = 0*.*0001* (Fig 5).

### Risk factors associated with transfusion-transmissible infections among blood donors

The study analyzed risk factors associated with transfusion-transmissible infections in blood donors. Male gender was significantly associated with HBV infection (OR = 1.81, 95% CI: 1.7–2.0, *P = 0*.*0001*) and female gender was significantly associated with infection to HIV (OR = 1.89, 95% CI: 1.7–2.2, *P = 0*.*0002*). Age groups between 35–44 and ≥ 45 years were significantly associated with HIV infections (OR = 1.73, 95% CI: 1.5–2.0, *P = 0*.*0002* and OR = 1.34, 95% CI: 1.01–1.8, *P = 0*.*038* respectively). For HBV infection, age groups between 18–24, 25–34 and 35–44 years were significantly associated (OR = 1.21, 95% CI: 1.03–1.4, *P = 0*.*017*; OR = 1.57, 95% CI: 1.3–1.8, *P = 0*.*0001* and OR = 1.51, 95% CI: 1.3–1.8, *P = 0*.*0001* respectively). Family or replacement blood donors were significantly associated with HIV infection (OR = 1.34, 95% CI: 1.2–1.5, *P = 0*.*0002*) and with HBV infection (OR = 1.24, 95% CI : 1.2–1.3, *P = 0*.*0001*) (Table 3). Age groups between 35–44 and ≥ 45 years were significantly associated with HCV infection (OR = 1.25, 95% CI: 1.0–1.5, *P = 0*.*038* and OR = 1.41, 95% CI: 1.0–2.0, *P = 0*.*047* respectively). For Syphilis infection, age groups between 25–34, 35–44 and ≥ 45 years were significantly associated (OR = 1.43, 95% CI: 1.2–1.7, *P = 0*.*0001*; OR = 3.52, 95% CI: 2.9–4.2, *P = 0*.*0001* and OR = 7.71, 95% CI: 6.3–9.5, *P = 0*.*0001* respectively). But also family or replacement blood donors were significantly associated with HCV infection (OR = 2.88, 95% CI: 2.3–3.7, *P = 0*.*0001*) (Table 4).

**Table 3:** HIV and HBV seroreactivity according to socio-demographic data.

Variables		HIV					HBV				
		Number*	Positive (%)*	OR	95% CI	P-value	Number*	Positive (%)*	OR	95% CI	*P-value*
**Sex** Male^1^		30,872	858 (2,8)	-	0,026–0,029	*0*.*0002*	59,709	4,735 (7.9)	1.81	1.7–2.0	*0*.*0001*
Female		6,327	333 (5,3)	1.89	1.7–2.2		18,083	794 (4.4)	-	0.04–0.05	
**Age** 18–24		12,324	328 (2,7)	-	0.24–0.03	*-*	29,068	1,781 (6.1)	1.21	1.03–1.4	*0*.*017*
	25–34 35–44	16,679	501 (3,0)	1.12	0.98–1.3	*0*.*093*	33,561	2,679 (8.0)	1.57	1.3–1.8	*0*.*0001*
		6,549	303 (4,6)	1.73	1.5–2.0	*0*.*0002*	11,702	894 (7.6)	1.51	1.3–1.8	*0*.*0001*
	≥45	1,647	59 (3,6)	1.34	1.01–1.8	*0*.*038*	3,461	175 (5.1)	-	0.04–0.06	*-*
**Type of blood donor** Volunteer^1^ Family		9,599	245 (2,6)	-	0.023–0.029	*0*.*0002*	25,980	1,592 (6.1)	-	0.06–0.064	*0*.*0001*
		27,600	946 (3,4)	1.34	1.2–1.5		51,812	3,937 (7.6)	1.24	1.2–1.3	

*Studies that provided data on HIV and HBV seroreactivity according to sociodemographic data have been included in this table.

^**1**^Group of reference; **OR** : Odds Ratio **; 95% CI**: 95% Confidence Interval.

**Table 4:** Seroreactivity of Syphilis and HCV according to socio-demographic data.

Variables		Number*	Syphilis				HCV				
			Positive (%) *	OR	95% CI	P-value	Number*	Positive (%) *	OR	95% CI	P-value
**Sex** Male		27,959	909 (3,3)	1.10	0.89–1.3	*0*.*497*	27,959	593 (2.1)	1.13	0.91–1.38	*0*.*269*
Female^1^		5,571	171 (3,1)	-	0.027–0.036		5,571	105 (1.9)	*-*	0.016–0.023	
**Age** 18–24^1^		11,689	198 (1,7)	-	0.015–0.019	*-*	11,689	229 (1.95)	*-*	0.017–0.022	*-*
	25–34 35–44	14,679	355 (2,4)	1.43	1.2–1.7	*0*.*0001*	14,679	289 (1.96)	1.01	0.8–1.2	*0*.*99*
		5,753	343 (5,96)	3.52	2.9–4.2	*0*.*0001*	5,753	141 (2.5)	1.25	1.0–1.5	*0*.*038*
	≥45	1,409	184 (13,1)	7.71	6.3–9.5	*0*.*0001*	1,409	39 (2.8)	1.41	1.0–2.0	*0*.*047*
**Type of blood donor** Volunteer^1^ Family		8,737	266 (3,0)	-	0.03–0.034	*0*.*292*	8,737	76 (0.9)	-	0.01–0.011	*0*.*0001*
		24,793	814 (3,3)	1.07	0.9–1.2		24,793	622 (2.5)	2.88	2.3–3.7	

^**1**^Group of reference; **OR** : Odds Ratio **; 95% CI** : 95% Confidence Interval.

*Studies that provided data on HCV and syphilis seroreactivity according to sociodemographic data have been included in this table.

## Discussion

Donating blood in hospitals saves lives, especially during surgery or in cases of severe anemia in children and pregnant women. But blood transfusion also constitutes a risk of transmitting transfusion-transmissible infections, in particular HIV, HBV, HCV and syphilis **[34]**. These TTIs are constantly encountered in transfusion circles in the world in general and in Gabon in particular. In this review, male blood donors (78.1%) and the age group between 25–34 (44.5%) were more frequent blood donors. This observation could be explained by the fact that young people are more motivated to donate blood for a simple reason which is free screening for TTIs after having adopted risk behaviors (unprotected sexual intercourse, tattooing, drug injection, etc.) exposing them to any contamination. But the low attendance of women would be due to their physiological state (pregnancy, menstruation). Some studies carried out in Africa and the rest of the world, notably in Cameroon (85.20%), India (90.70%), Iran (96.79%) and Nigeria (98.70%) have shown that younger men were more active in blood donation **[35–38]**. The combined prevalence of TTIs among blood donors namely HIV, HBV, HCV and syphilis was 3.0%, 6.0%, 4.0% and 3.0% respectively. The combined prevalence of HIV was high compared to the prevalences obtained in meta-analyses carried out in Iranian blood donors (0.0079%) **[39]** and Ethiopian blood donors (2.69%) **[34]**. Heterogeneity of included studies for combined HIV prevalence was high (I^2^ = 98.5%, *P = 0*.*0001*). Possible reasons for this difference could be attributed to the algorithms and test kits used to diagnose HIV. But also the type of blood donors (voluntary and family) could also influence this variation observed in these different studies carried out in these different countries. However, family blood donors are more at risk, which could also justify this difference in combined prevalence. Some studies conducted on blood transfusion in some countries have shown that the prevalence of HIV is high, hence the need to strengthen current blood donor selection and screening algorithms **[40–45]**. Similarly, the combined prevalence of HBV was high compared to the prevalences obtained in systematic reviews carried out in blood donors Eastern Mediterranean and Middle Eastern countries (2.03%) **[46]** and in Ethiopian blood donors (4.91%) **[47,48]**. The heterogeneity of the included studies for the combined prevalence of HBV was high (I^2^ = 99.3%, *P = 0*.*0001*). This could be explained by prevention practices against TTIs which differ from one country to another but also the financial situation of the populations making them vulnerable to any contamination with sexually transmitted infections. In addition, the discrepancy could also be due to the donation screening strategies used in the different blood banks. Note that in several studies conducted in other countries revealed that the prevalence of HBV was high among blood donors and this requires corrective measures to reduce the prevalence of TTIs including HBV **[42,49–52]**.

The combined prevalence of HCV was higher compared to the prevalence obtained in study carried out in Ethiopia (0.819%) **[53].** It was similar to that obtained in the meta-analysis conducted in Democratic Republic Congo (5.0%) **[54]**. It should also be noted that Egypt has the highest prevalence of HCV (14.5%) in the world **[55,56]**. The heterogeneity value of the included studies for HCV prevalence was high (I^2^ = 99.75%, *P = 0*.*001*). This gap between the different prevalences could be justified by the non-application of preventive measures against TTIs but also due to screening strategies that sometimes differ from one country to another. Some authors have shown that the prevalence of HCV in blood donors is worrying **[42,57–60]**. The combined prevalence of syphilis was higher compared to prevalences obtained in studies carried out among Ethiopian blood donors (1.50%) **[34]** and Iranian blood donors (0.1%) **[61].** The value of heterogeneity of the included studies for syphilis was high (I^2^ = 93.6%, *P = 0*.*0001*). This finding could be explained by the different diagnostic methods used by each country in blood banks. But also by the blood donor selection methods which differ from one blood bank to another or from one country to another. Several studies conducted in transfusion settings in different countries have shown that the prevalence of syphilis is high **[62–66]**. The female gender was more at risk and significantly associated with HIV infection, whereas the male gender was more at risk and significantly associated with HBV infection. Blood donors aged 35 and over were at greater risk and significantly associated with HIV, HCV and Syphilis infections. On the other hand, blood donors aged 18 to 44 were more at risk and significantly associated with HBV infections. Family donors were more at risk and significantly associated with HIV, HBV and HCV infection. These results could be explained by the fact that blood donors aged 18 to 44 are the most sexually active and are exposed to any contamination with a sexually transmitted infection (HIV, HCV, HBV and syphilis) because the majority of these young people do not respect prevention measures during sexual intercourse. Indeed, several authors have shown that sex and age were factors associated with TTIs. For example, studies conducted in Egypt, Malawi and India which showed that young people were more exposed than other categories of blood donors **[67–70]**. Note also that blood donor type was also significantly associated with TTIs. According to the WHO, family blood donors are the most at risk of transmitting TTIs and moreover they are made up mostly of young people whose age varies between 18 and 35. This would also be explained by the age group where sexual activity is intense and often leads to the adoption of risk behavior, that is to say the absence of preventive measures (wearing a condom for example). These data corroborate those obtained in several studies carried out in certain regions of Africa notably in Ethiopia and in other regions of the world notably in India and Pakistan **[67,71–73]**. To reduce the infection rate among blood donors, it would be necessary to strengthen rigorous blood donor selection strategies in order to exclude at-risk blood donors. And regarding blood donors at higher risk, namely family donors, blood bank managers must initiate awareness campaigns to further motivate voluntary donors to give blood. In addition, not to condition the acquisition of a blood bag by compensation donations because this would promote a supply of family blood donations, that is to say donations at risk. In the case of hepatitis B, it would also be necessary for health authorities to step up vaccination campaigns in order to also reduce the rate of HBV infection in the general population and above all. in blood donors.

### Strengths and limitations

This systematic review and meta-analysis study is, to our knowledge, the first systematic review and meta-analysis estimating the combined prevalence of transfusion-transmissible infections among blood donors in Gabon. Additionally, the included studies were only from areas of the country where the prevalence of infection-borne infections has been documented. However, we did not find an evident reduction in heterogeneity after using the random effects model of the pooled prevalences of the included studies. But unfortunately, this significance of the heterogeneity of the combined prevalence can be influenced by the sample size. Also the number of studies included in the systematic review can also favor a deviation of the inference.

## Conclusion

This systematic review and meta-analysis showed that the combined prevalence of TTIs was high among Gabonese blood donors. To reduce the risk of TTIs and ensure transfusion safety, it is necessary to respect the selection criteria, the blood donor selection algorithm and the use of very sensitive and specific techniques for the detection of TTIs. Furthermore, prevention of TTIs among blood donors should be strengthened with emphasis on public awareness campaigns. Blood bank managers should also implement all applications to improve donation safety strategies in a comprehensive approach to TTI monitoring to reduce this burden.

## Supporting information

S1 Checklist PRISMA 2020 checklist. (PDF)

S1 Fig Funnel plot of HIV studies. (TIF)

S2 Fig Funnel plot of HBV studies. (TIF)

S3 Fig Funnel plot of HCV studies. (TIF)

S4 Fig Funnel plot of Syphilis studies. (TIF)

S5 Fig Egger’s publication bias plot of HIV studies. (TIF)

S6 Fig Egger’s publication bias plot of HBV studies. (TIF)

S7 Fig Egger’s publication bias plot of HCV studies. (TIF)

S8 Fig Egger’s publication bias plot of Syphilis studies. (TIF)

S1 Table Search strategy for Pubmed, Embase and Google Scholar. (DOCX)

S2 Table PRISMA 2020 checklist. (PDF)

S3 Table Assessment of methodological quality of included articles. (DOCX)

S4 Table Scoring criteria for quality of studies (Adapted from Stanifer et al.). (DOCX)
