# Supplementary figures and images for: Prevalence and factors associated with transfusion-transmissible infections (HIV, HBV, HCV and Syphilis) among blood donors in Gabon: Systematic review and meta-analysis
Source: PLoS One. 2024 Aug 19;19(8):e0307101. doi: 10.1371/journal.pone.0307101 (PMC11332953; doi:10.1371/journal.pone.0307101)

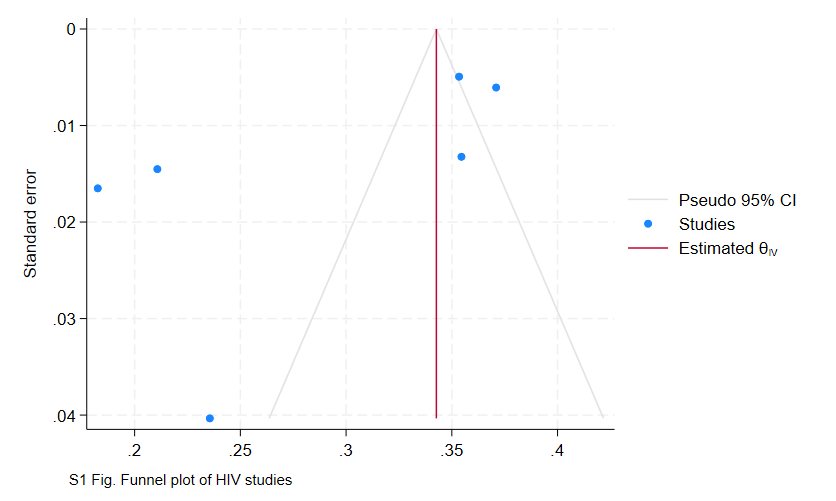

Supplement: S1 Fig — (TIF) [file pone.0307101.s002.tif]

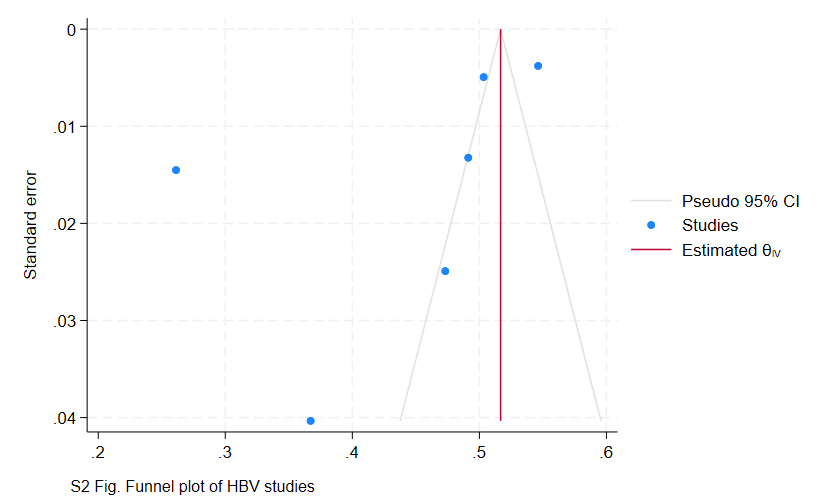

Supplement: S2 Fig — (TIF) [file pone.0307101.s003.tif]

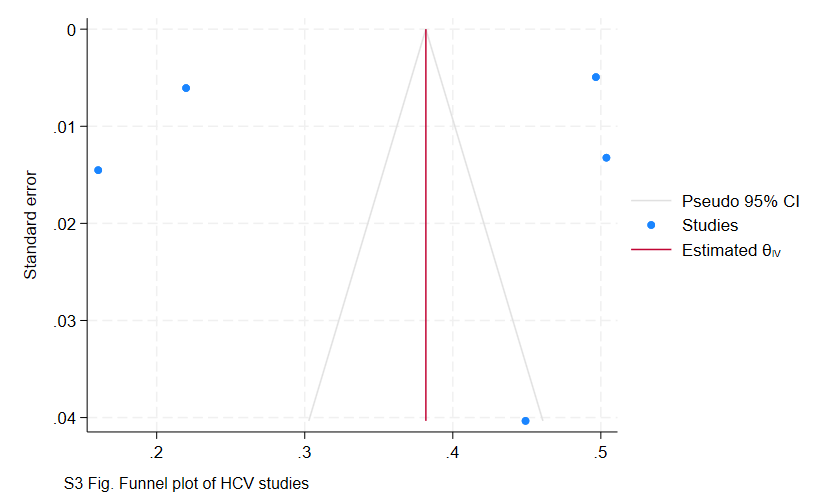

Supplement: S3 Fig — (TIF) [file pone.0307101.s004.tif]

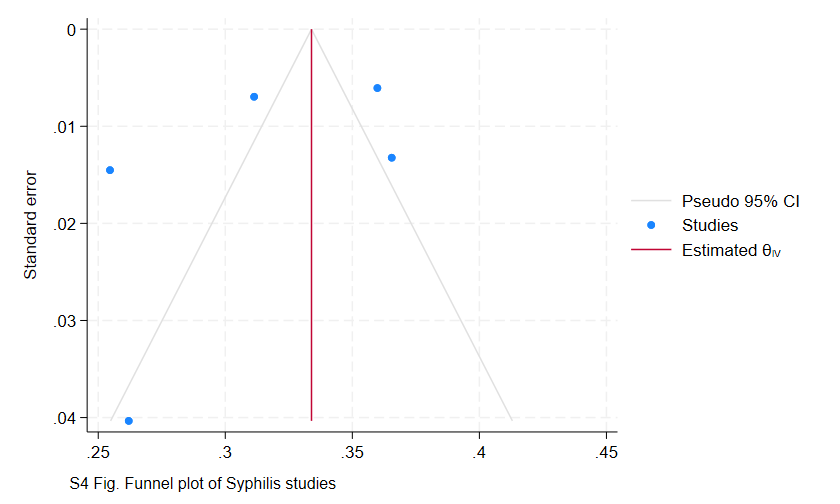

Supplement: S4 Fig — (TIF) [file pone.0307101.s005.tif]

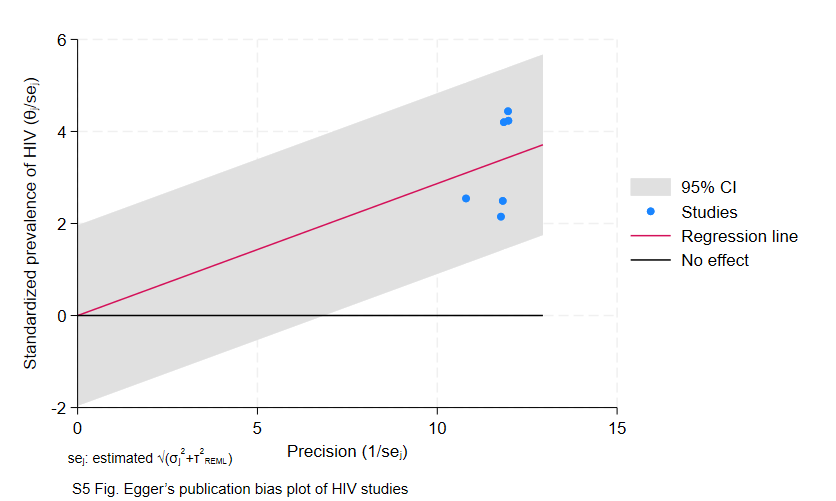

Supplement: S5 Fig — (TIF) [file pone.0307101.s006.tif]

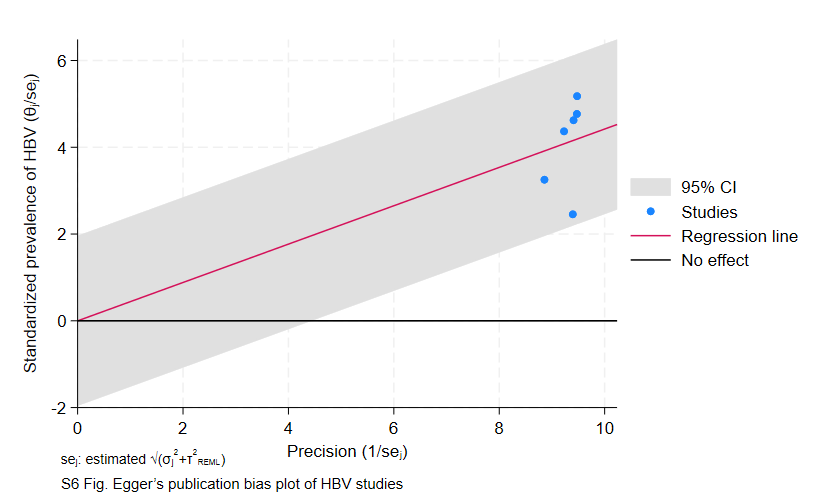

Supplement: S6 Fig — (TIF) [file pone.0307101.s007.tif]

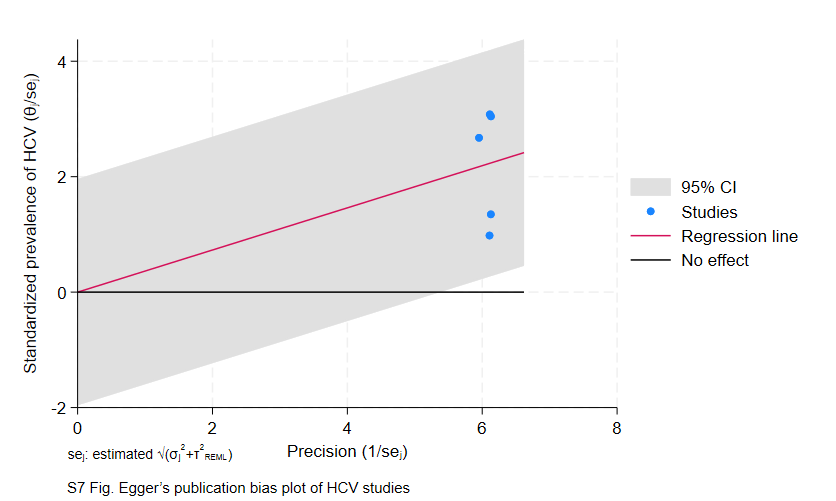

Supplement: S7 Fig — (TIF) [file pone.0307101.s008.tif]

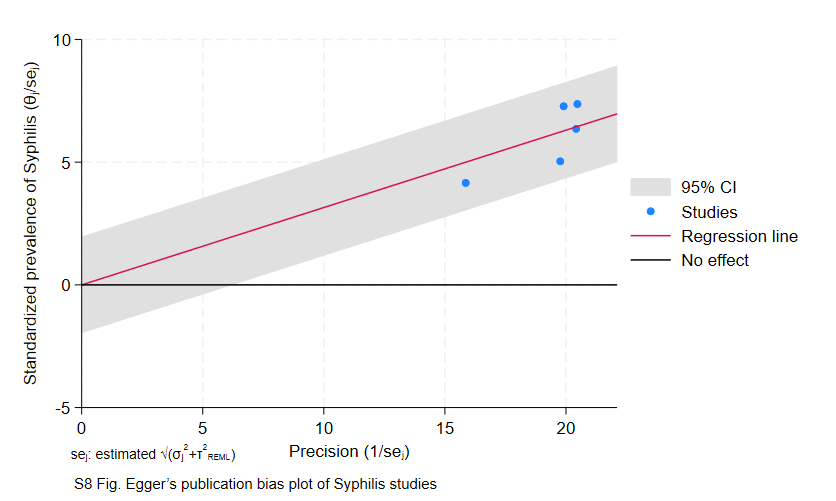

Supplement: S8 Fig — (TIF) [file pone.0307101.s009.tif]
