# Supplementary material for: Prevalence and factors associated with transfusion-transmissible infections (HIV, HBV, HCV and Syphilis) among blood donors in Gabon: Systematic review and meta-analysis
Source: PLoS One. 2024 Aug 19;19(8):e0307101. doi: 10.1371/journal.pone.0307101 (PMC11332953; doi:10.1371/journal.pone.0307101)
Supplement: S1 Table — (DOCX) [file pone.0307101.s010.docx]

**S1 Table. Search strategy for Pubmed, Embase and Google Scholar**

| **Search** | **Bibliographic search equations** |
| --- | --- |
| **#1** | «Prevalence, HIV, HBV, HCV, syphilis, blood donors, Gabon» |
| **#2** | « Prevalence, transfusion-transmitted infections, Gabon » |
| **#3** | «Prevalence, HIV, HBV, HCV, syphilis, factors, transfusion, Gabon » |
| **#4** | Publication period: 01/01/2014 to 01/12/2022 |
