# Supplementary material for: Prevalence and factors associated with transfusion-transmissible infections (HIV, HBV, HCV and Syphilis) among blood donors in Gabon: Systematic review and meta-analysis
Source: PLoS One. 2024 Aug 19;19(8):e0307101. doi: 10.1371/journal.pone.0307101 (PMC11332953; doi:10.1371/journal.pone.0307101)
Supplement: S3 Table — (DOCX) [file pone.0307101.s012.docx]

**S3 Table.** **Assessment of methodological quality of included articles**

| **Author name** | **Study design** | **Q1** | **Q2** | **Q3** | **Q4** | **Q5** | **Q6** | **Q7** | **Q8** | **Q9** | **Total** | **Quality** |
| --- | --- | --- | --- | --- | --- | --- | --- | --- | --- | --- | --- | --- |
| Rerambiah et al 2014 A (1) | Retrospective | 1 | 1 | 1 | 1 | 1 | 1 | 0 | 0 | 1 | 7 | High |
| Bisseye et al 2017 (2) | Retrospective | 1 | 1 | 0 | 0 | 0 | 1 | 0 | 0 | 0 | 3 | Low |
| Tonda et al 2017 (3) | Retrospective | 1 | 1 | 1 | 1 | 1 | 1 | 1 | 0 | 1 | 8 | High |
| Eko Mba et al 2017 (4) | Retrospective | 1 | 1 | 1 | 1 | 1 | 1 | 1 | 0 | 1 | 8 | High |
| Bisseye et al 2018 (5) | Retrospective | 1 | 1 | 1 | 1 | 1 | 1 | 1 | 0 | 1 | 8 | High |
| Bisseye et al 2019 (6) | Retrospective | 1 | 1 | 1 | 1 | 1 | 1 | 1 | 1 | 0 | 8 | High |
| Ngassaki-Yoka et al 2018 (7) | Retrospective | 1 | 1 | 1 | 1 | 1 | 1 | 1 | 0 | 1 | 8 | High |
| Mangala et al 2021 (8) | Cross-sectional | 1 | 1 | 1 | 1 | 1 | 1 | 1 | 1 | 0 | 8 | High |
| Maulot-Bangola et al 2021 (9) | Cross-sectional | 1 | 1 | 1 | 1 | 1 | 1 | 1 | 1 | 0 | 8 | High |
| Rerambiah et al 2014 B (10) | Retrospective | 1 | 1 | 1 | 0 | 0 | 1 | 0 | 0 | 1 | 5 | Medium |
| Eko Mba et al 2018 (11) | Retrospective | 1 | 1 | 1 | 1 | 1 | 1 | 1 | 1 | 0 | 8 | High |
| Ramassamy et al 2020 (12) | Cross-sectional | 1 | 1 | 0 | 1 | 1 | 1 | 0 | 0 | 0 | 5 | Medium |
| Kenguele et al 2020 (13) | Cross-sectional | 1 | 1 | 0 | 1 | 0 | 1 | 0 | 0 | 0 | 4 | Medium |

**High** : 7-9, **Medium** : 4-6, **Low**: less than 4, **Q1** : question N°1
