# Supplementary material for: Prevalence and factors associated with transfusion-transmissible infections (HIV, HBV, HCV and Syphilis) among blood donors in Gabon: Systematic review and meta-analysis
Source: PLoS One. 2024 Aug 19;19(8):e0307101. doi: 10.1371/journal.pone.0307101 (PMC11332953; doi:10.1371/journal.pone.0307101)
Supplement: S4 Table — (DOCX) [file pone.0307101.s013.docx]

**S4 Table.** **Scoring criteria for quality of studies (Adapted from Stanifer et al.)**

| **N°** | **Assessment criteria questions** | **Yes= 1** | **No = 0** |
| --- | --- | --- | --- |
| 1 | Is sample size greater than or equal to 300 (≥300 is considered appropriate size)? |  |  |
| 2 | Are study participants donors? |  |  |
| 3 | Has the prevalence of any of the four TTIs been determined? |  |  |
| 4 | Are socio-demographic data available? |  |  |
| 5 | Is the test used for screening donations serological 3rd or 4th generation)? |  |  |
| 6 | Was the study conducted in a Gabonese transfusional settings? |  |  |
| 7 | Has the seroreactivity of TTIs according to socio-demographic data been determined? |  |  |
| 8 | Has statistical analysis for the determination of factors associated with TTIs been carried out? |  |  |
| 9 | Has the prevalence of three to four TTIs been determined? |  |  |

**High quality**: 7-9, **Medium quality**: 4-6, **Low quality**: less than 4
